# Glial Fibrillary Acidic Protein Astrocytopathy in Pediatric Patients: A Retrospective Study

**DOI:** 10.3389/fped.2020.626564

**Published:** 2021-01-25

**Authors:** Haixia Huang, Ke Bai, Yueqiang Fu, Siwei Lu, Yunni Ran, Hongxing Dang, Jing Li

**Affiliations:** ^1^Intensive Care Unit, Key Medical Laboratory of Pediatrics, Chongqing Health Bureau, Ministry of Education, Chongqing, China; ^2^Key Laboratory of Child Development and Disorders, Children's Hospital of Chongqing Medical University, Chongqing, China

**Keywords:** child, glial fibrillary acidic protein astrocytopathy, meningoencephalitis, myelitis, steroids

## Abstract

Autoimmune glial fibrillary acidic protein astrocytopathy is a novel form of autoimmune meningoencephalitis related to GFAP autoantibodies. This condition is still being characterized, and few pediatric patients have been identified. Here, we report three patients presenting with fever, nausea, and headache, following progressive disturbance of consciousness, limb weakness, dyspnea, or urine retention. MRI analysis revealed that T2-hyperintense lesions, or enhancement of the meninges and spinal cord. CSF and serum analyses revealed they were positive for GFAP antibody, confirming GFAP astrocytopathy diagnosis. Treating the patients with IVIG, with or without intravenous steroids, gradually improved their clinical symptoms. Our findings indicate that GFAP astrocytopathy should be considered in children who are clinically diagnosed with meningoencephalitis, whether or not myelitis is present, and if the MRI reveals enhancement of meninges or spinal cord, T2-hyperintense lesions, or a pattern of linear perivascular gadolinium enhancement. Suspected cases should be tested for GFAP antibody as soon as possible because these patients may benefit from immunotherapy.

## Introduction

Glial fibrillary acidic protein (GFAP) astrocytopathy is a recently identified type of autoimmune meningoencephalitis related to GFAP autoantibodies. Its clinical presentations include encephalopathy, headache, myelopathic symptoms, abnormal vision/optic neuritis, postural tremor, and cerebellar ataxia. GFAP astrocytopathy has almost exclusively been described in adults (1–4), with very few reported pediatric cases. Thus, its clinical features in children remain to be defined. Here, we present three pediatric cases of GFAP astrocytopathy and provide a literature review regarding this novel disorder.

## Methods

Between January 2020 and October 2020, three pediatric patients were diagnosed with GAFP astrocytopathy at the Children's Hospital of Chongqing Medical University, the largest tertiary children's medical center in southwest China. The patients were subjected to detailed clinical examination, routine cerebrospinal fluid (CSF) examination, and magnetic resonance imaging (MRI) analysis. Serum and CSFs were examined for GFAP IgG using a cell-based assay that relies on HEK293 cells co-transfected with human GFAP and pEGFP-N1. Thirty-six hours after transfection, the cells were fixed with 4% paraformaldehyde (PFA) for 20 min and permeabilized with 0.1% Triton X-100 in 1X phosphate buffered saline (PBS) for 20 min. They were then incubated with patient's CSF or blood for 2 h and then immunolabeled with an AlexaFluor 546 secondary antibody against human IgG (Thermo Scientific) diluted at 1:1000 for 1 h at room temperature and imaged on a Zeiss Axiovert A1 fluorescence microscope.

Ethical approval for the study was granted by the ethics committee of the Children's Hospital of Chongqing Medical University. Written informed consents were obtained from the patients' parents or legal guardians.

## Results

**Figure 1:**
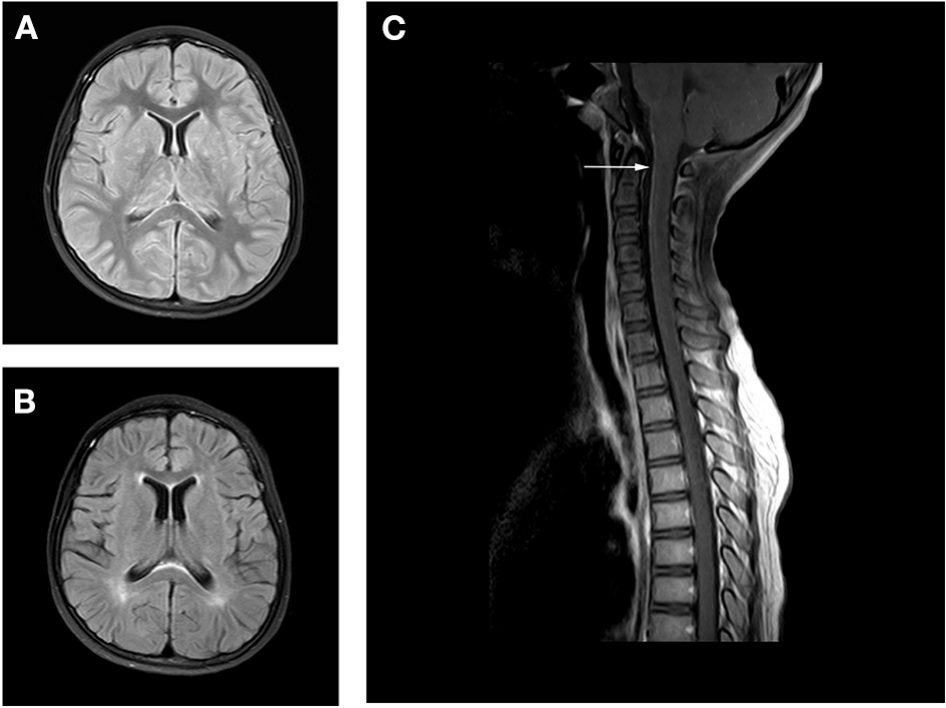
Axial fluid attenuation inversion recovery (FLAIR) MRI brain **(A)** upon admission and **(B)** 2 months later. Image **(A)** showed T2-hyperintense lesions in in bilateral thalamus and basal ganglia. Follow-up image **(B)** showed improved T2 lesions. MRI of the spine **(C)** 10 days after admission showed leptomeningeal enhancement in the cervical cord.

**Figure 2:**
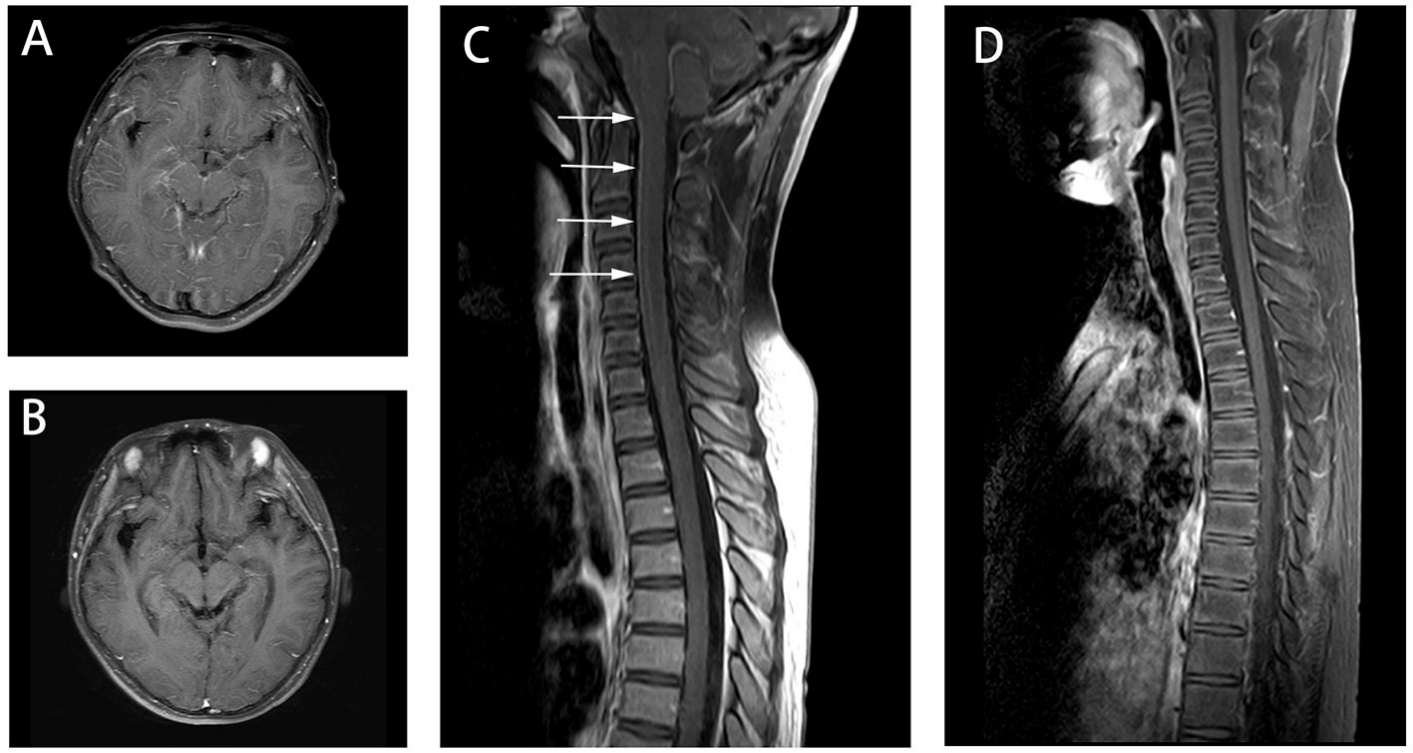
Axial fluid attenuation inversion recovery (FLAIR) MRI brain **(A)** 7 days after admission and **(B)** 3 weeks later. Image **(A)** showed the enhancement in the surface of the large and small brain as well as brainstem (not shown). Image **(B)** showed meningeal enhancement was reduced. Spine MRI **(C)** 10 days after admission and **(D)** 3 weeks later. Image **(C)** showed the enhancement of spine. And Image **(D)** showed the enhancement was reduced after the immunotherapy treatment.

**Figure 3:**
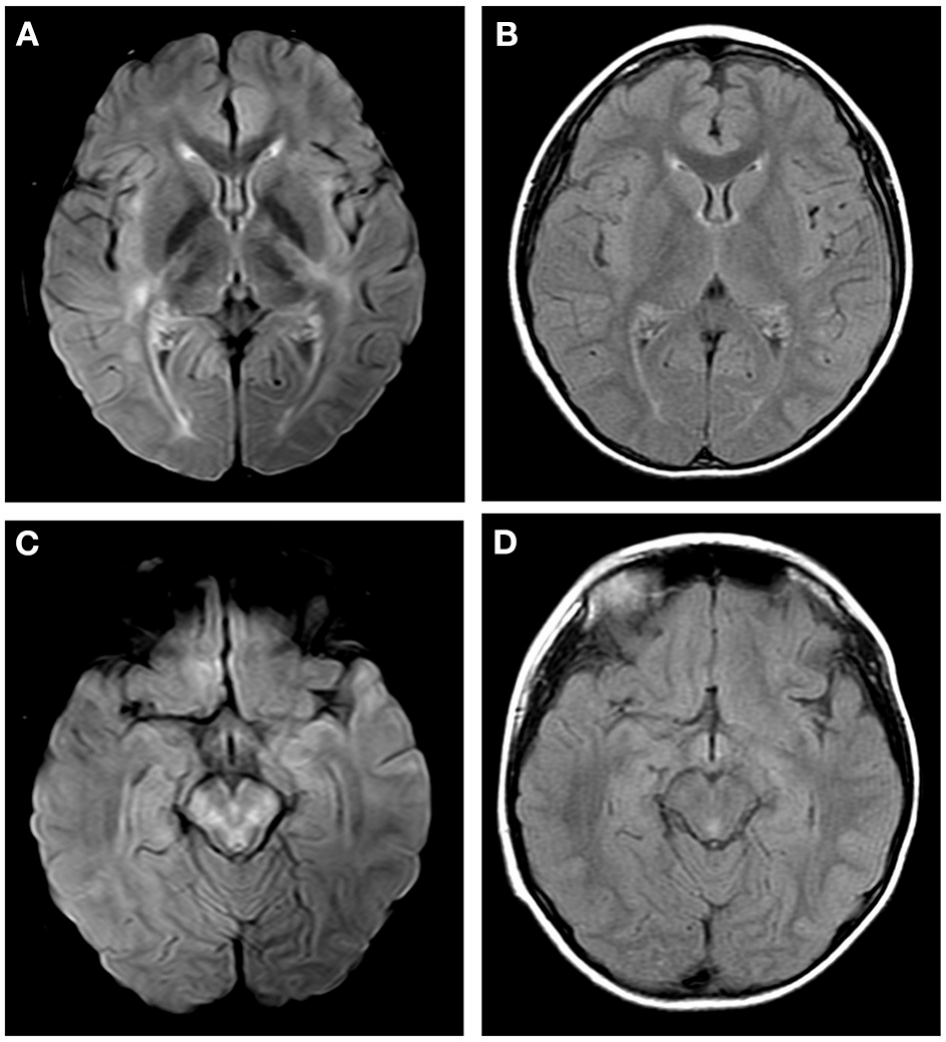
Axial fluid attenuation inversion recovery (FLAIR) MRI brain **(A,C)** 12 days after admission and **(B,D)** 1 month later. Image **(A)** showed T2-hyperintense lesions in internal capsule, hind limbs and external capsule. Image **(C)** showed T2-hyperintense lesions in brain stem. Follow-up images **(B,D)** showed improved T2 lesions.

**Figure 4:**
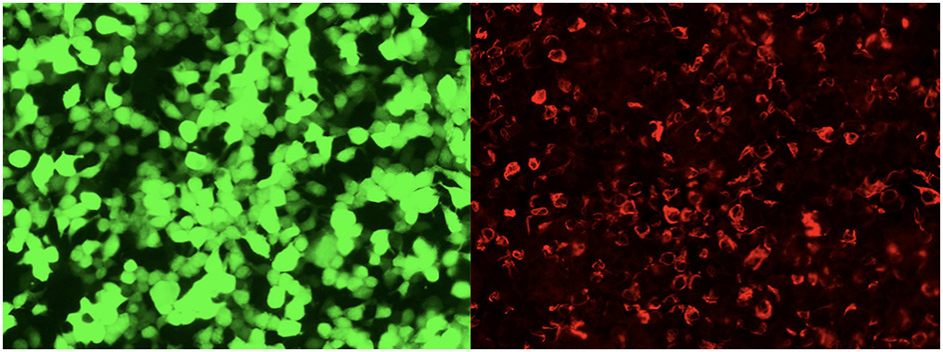
The glial fibrillary acidic protein autoantibodies in the cerebrospinal fluid of the patients were transfected into cell-indirect immunofluorescence detection: human embryonic kidney 293T (HEK293T), after cell fixation and permeabilization, immunofluorescence detection of GFAP autoantibodies in the sample. In the picture, green is the cells transfected to express GFAP, and red is human IgG that binds to the corresponding antigen.

All patients (1 girl and 2 boys) were adolescent and presented with flu-like symptoms, including fever, nausea, and headache lasting 5–10 days. They then progressively developed disturbed of consciousness and urine retention. Patients 1 and 2 also developed dyspnea and limb weakness. The patients were initially diagnosed with infectious meningoencephalitis, with or without myelitis, and treated with antibiotics and antivirals, but their conditions did not improve. Tests for various infections, including mycoplasma, Epstein-Barr virus, herpes simplex virus, coxsackie virus, measles virus, influenza virus, mycobacterium tuberculosis, and enterovirus returned normal or negative results. In all the cases, white blood cell count, neutropenia, lymphocytes, and C-reactive protein were normal. However, procalcitonin was slightly elevated at 0.06–0.895 ng/mL (normal reference value: < 0.05). Proof of intracranial infection was insufficient. In the course of the disease, patients experienced hallucinations or blurred vision. Lumbar puncture revealed elevated leukocytes and protein levels, and normal glucose levels. Thus, immune factors or demyelinating diseases were suspected. MRI analysis of Patient 1 revealed T2-hyperintense lesions in bilateral thalamus and basal ganglia, and partial spinal enhancement in the cervical cord (Figures 1A,C). Patient 2 brain MRI did not find obvious abnormalities in the brain parenchyma, although enhancement was observed on large and small brain surfaces and the brainstem (Figures 2A,C). The nerve roots, cauda equina, and terminal filaments were also linearly strengthened. Patient 3 brain MRI revealed T2-hyperintense lesions (Figures 3A,C). However, the spinal MRI was normal. Based on these imaging data, we analyzed the patients' CSF and blood for the presence of GFAP autoantibodies and found them positive in both CSF and blood, suggesting a diagnosis of GFAP astrocytopathy (Figure 4). Analyses of MOG/NMDA/AQP-4 presence in the patients CSF and blood returned negative results. However, antinuclear antibody and double-stranded DNA antibodies were detected in Patient 1. Tumor markers and enhanced computed tomography of the chest and abdomen revealed no apparent abnormalities in Patients 1 and 2, who are under continued monitoring. This analysis was not done on Patient 3.

Following confirmation of GFAP astrocytopathy in Patients 1 and 2, immunotherapy was prescribed and immunoglobulin (1 g/kg/day) administered intravenously for 2 days. The patients also received intravenous methylprednisolone (15–20 mg/kg/day) for 5 days. They were then prescribed long-term oral prednisone (1.5–2 mg/kg/day). Patient 3 was treated with intravenous immunoglobulin (1 g/kg/day) only, for 2 days. All patients gradually improved upon treatment. Follow-up brain MRI on Patient 1 revealed that T2-hyperintense lesions had been improved (Figure 1B). However, a follow-up spinal cord MRI was not done. MRI on Patient 2 revealed reduced enhancement and that nerves, cauda equina, and terminal filaments were not significantly strengthened (Figures 2B,D). MRI on Patient 3 revealed improved T2-hyperintense lesions (Figures 3B,D).

## Discussion

GFAP is an intermediate astrocyte protein, between the smaller microfilaments and larger microtubules. It is a target and biomarker for astroglial pathology in neurological diseases. GFAP is associated with a range of neurological disorders, including central nervous system (CNS) immune inflammatory diseases, congenital diseases, and traumatic disorders. Autoimmune GFAP astrocytopathy is a recently defined autoimmune disease of the CNS (5) and is characterized by vision abnormalities as well as meningeal, encephalitic, and myelitic symptoms. A specific IgG autoantibody has been identified in patients with a CNS syndrome recently defined as autoimmune GFAP astrocytopathy. However, very few pediatric cases have so far been reported, with the youngest known case involving a 6-year-old.

A review of English and Chinese literature, using the search term “GFAP astrocytopathy” identified only eight articles describing pediatric cases of GFAP astrocytopathy involving a total of 18 children with serum and/or CSF GFAP-IgG (3, 4, 6–11). However, one patient was not described in detail. Only four pediatric cases were GFAP-IgG positive in serum only, and their clinical manifestations and imaging analysis did not meet the diagnosis criteria for GFAP astrocytopathy. Thus, the four were excluded from the study. Data on the three present cases of GFAP astrocytopathy and previously published ones are summarized in Table 1. Of these, 10 cases were male and 6 were female, with the oldest aged 15 and the youngest aged 6. Fifteen patients (93.75%) presented with meningoencephalitis. Six cases (40%) also had myelitis, and one (6.6%) also had blurred vision, indicating that in children, meningoencephalitis (with/without myelitis) is the disorder's most common clinical feature, which is consistent with adult patient data. CSF analysis from 15 patients showed that 13/15 (86.6%) had inflammatory CSF characterized by elevated protein and white blood cells in the CSF. A normal MRI is rare in adult GFAP astrocytopathy. Approximately half of affected patients had hyperintense lesions on T2-weighted sequences, and > 50% of the patients had a characteristic linear, radial perivascular pattern of enhancement through the cerebral white matter, emanating from GFAP-enriched peri-lateral ventricular regions (1). While these findings are not pathognomonic, they aid diagnosis considerably. Twelve of these 16 patients (75%) had abnormal MRIs. However, the most common MRI (eight cases, 50%) in pediatric cases was meninges or spine enhancement. T2-hyperintense lesions were present in five cases (31.3%). Only one case (6.25%) had a periventricular radial linear enhancement, which is markedly lower than in adults. Determination of whether there are MRI differences in adults and children is limited by scarcity of known pediatric cases.

**Table 1:** Summary of pediatric patients' clinical characteristics.

**Case No (Ref)**	**Age/sex**	**Clinical diagnosis**	**MRI**	**CSF Finding**	**GFAP IgG**	**Immunomodulatory therapy**	**Response to therapy**
					**CSF/Serum**		
Patient 1	F/10	Meningoencephalomyelitis	Leptomeningeal enhancement of the cervical spine,T2-hyperintense lesions in basal ganglia	Protein 90 mg/dl, TNC∧: 136/μl	+/+	IVMP, IVIG	Favorable^*^
Patient 2	M/13	Meningoencephalomyelitis	The enhancement of meninges and spinal cord, the nerve roots were linearly strengthened.	Protein 77 mg/dl, TNC∧: 334/μl	+/+	IVMP, IVIG	Favorable^*^
Patient 3	M/7	Meningoencephalitis	T2-hyperintense lesions in basal ganglia and brain stem	Protein 84 mg/dl, TNC∧: 110/μl	+/+	IVIG	Favorable^*^
Li	M/11	Meningoencephalitis	Meningeal enhancement, T2-hyperintense lesions in basal ganglia	Protein 171 mg/dl, TNC∧: 169/μl	+/NA	IVMP, IVIG	Favorable^*^
Lorio	M/6	Encephalitis	T2-hyperintense lesions in temporal, parietal, frontal cortex	Normal	+/+	IVIG	Favorable^*^
Dubey 1	F/8	Meningoencephalitis, aphasia	Meningeal enhancement	protein 55 mg/dl, TNC∧: 159/μl	+/–	IVMP, PLEX, Rituximab	Favorable^**^
Dubey 2	F/10	Meningoencephalitis	meningeal and patchy parenchymal enhancement	Protein 47 mg/dl, TNC∧: 71/ul	+/+	IVMP	Favorable^*^
Dubey 3	M/10	Menignoencephalomyelitis, autonomic dysfunction	meningeal and patchy parenchymal enhancement	Protein 60 mg/dl, TNC∧: 155/ul	+/–	IVMP	Favorable^*^
Dubey 4	M/12	Meningitis, myelopathy	Normal	Protein 211 mg/dl, TNC∧: 12/μl	+/NA	IVMP, IVIG	Favorable^*^
Dubey 5	M/13	Meningoencephalitis	Normal	Protein 287 mg/dl, TNC∧: 148/ul	+/NA	IVMP, IVIG	Favorable^*^
Dubey 6	M/15	Menignoencephalomyelitis	Normal	Protein 65 mg/dl, TNC∧: 51/μl	+/+	IVMP	Favorable^*^
Dubey 7	F/15	Menignoencephalitis	Parenchymal enhancement and T2/FLAIR hyperintensity in parietal lobe	Normal	+/+	IVMP, IVIG, Rituximab	Favorable^**^
Trau	M/10	meningoencephalomyelitis	Leptomeningeal enhancement; T2 hyperintensities of the cervical and thoracic spine with enhancement of the cervical spine	NA	NA/ +	IVMP, IVIG, PLEX	Favorable^*^
Liana	F/15	Encephalitis;blurry vision	Periventricular radial linear enhancement	Protein 109 mg/dl, TNC∧: 100/μl	+ /NA	IVMP, IVIG, PLEX	Favorable^*^
Handoko	M/12	Psychiatric symptoms after HSE	Sequelae of HSE without acute findings	Protein 97 mg/d, elevated cell count	+/+	IVMP, IVIG, mycophenolate mofetil	Limited improvement
Francisco	F/6	Meningoencephalomyelitis	Diffuse leptomeningeal enhancement of bifrontal and biparietal cortical sulci	Protein 128 mg/dl, TNC∧: 385/μl	+/NA	IVMP, IVIG	Favorable^*^

NA, not available; TNC, total count; CSF, cerebrospinal fluid; F, female; M, male;

* Response to first line immunomodulatory therapy (IVMP, IVIG, PLEX);

** *Response following administration of second-line immunomodulatory therapy (rituximab, mycophenolate mofetil, cyclophosphamide)*.

Of 102 Mayo Clinic patients, 41 (40%) had one or more coexisting antibodies in serum or CSF (4), with NMDA and AQP-4-IgG antibodies being most common. In Table 1, 4 of 16 (25.0%) cases had coexisting antibodies, which is consistent with adult patient data. Antinuclear antibody and double-stranded DNA antibodies were encountered in Chinese patients (Li's and our patient 1) (11). Dubey reported two pediatric patients with coexisting NMDA antibody. Coexisting antibodies were not detected in the other cases. In 2017, Flanagan reported that 35 of 102 patients (34%) had neoplasia, and 66% of the tumors were detected within 2 years of symptom onset, including ovarian teratoma, adenocarcinoma, and glioma (4). Thus, patients should be monitored for underlying neoplasms within 2 years of GFAP disease onset. However, we found that the four pediatric cases that underwent tumor surveillance turned out negative.

Currently, there is no standard treatment for GFAP astrocytopathy. During the acute phase, clinical and radiological improvements were revealed by corticosteroids, intravenous immunoglobulin (IVIG), or plasmapheresis (PLEX). However, 20–50% of the patients had relapsing disease courses (12). Currently, the treatments for refractory or relapsed cases include long-term oral glucocorticoids and immunosuppressants. Analysis of acute treatment data for all cases found that in the acute phase, the condition of two children who received IVIG only, improved. The remaining 14 cases were all treated with steroids. Of these, four received steroids only, seven also received IVIG, and one also received PLEX. Of the 16 patients, two received a combination of steroids, IVIG and PLEX. Of four patients who received steroids alone, three improved, with the other improving after receiving rituximab. Of the seven patients treated with steroids and IVIG, only one failed to improve and continued to struggle with cognitive deficits 10 months after his diagnosis of GFAP astrocytopathy and after extending monthly IVIG, mycophenolate mofetil and oral steroids. The patient who received steroids plus PLEX only got better after extending rituximab. The two patients (100%) treated with a combination of steroids, IVIG and PLEX, improved. One of the two cases did not improve with steroids and IVIG but significantly improved after receiving PLEX. Taken together, these data suggest that most pediatric patients respond well to steroids and IVIG. However, some did not improve after receiving PLEX or immunosuppressants such as rituximab or mycophenolate mofetil. Thus, it may be better if pediatric GFAP astrocytopathy patients are treated early with PLEX and immunosuppressants. Of the 15 patients, data on chronic treatment and follow-up was available for five patients. Long-term immunosuppression and oral steroids included the following, alone or in combination: mycophenolate mofetil and oral steroids (1), oral steroids only (2), and rituximab and oral steroids (2). Median follow-up duration was 12 months (range: 5–60 months). Most of the patients had good prognosis without relapse, and only one child exhibited residual neuropsychiatric symptoms. All treatments and outcome data were observational, and not from prospective, controlled studies. Thus, there is an urgent need to collect treatment and outcome data, as well as a standardized treatment plan.

In conclusion, GFAP astrocytopathy, a relatively new autoimmune disorder of the CNS, should be considered in children clinically diagnosed with meningoencephalitis (whether or not it presents with myelitis) and have MRIs that reveal meninges or spine enhancement, T2-hyperintense lesions, or a pattern of linear perivascular gadolinium enhancement. Our data suggest that GFAP antibody analysis should be done as soon as possible in suspected patients. At present, steroids and IVIG are used during the acute phase, and where improvement is not observed, PLEX or immunosuppressives can be administered. Most patients have good prognoses. Additionally, pediatric GFAP astrocytopathy patients should be screened and monitored for autoimmune diseases or neoplasms from 2 years after diagnosis.

## Data Availability Statement

The original contributions presented in the study are included in the article/supplementary materials, further inquiries can be directed to the corresponding author/s.

## Ethics Statement

The studies involving human participants were reviewed and approved by the ethics committee of the Children's Hospital of Chongqing Medical University. Written informed consent to participate in this study was provided by the participants' legal guardian/next of kin. Written informed consent was obtained from the minor(s)' legal guardian/next of kin for the publication of any potentially identifiable images or data included in this article.

## Author Contributions

HH, KB, YF, SL, YR, JL, and HD analyzed and interpreted the data. HH, JL, and HD wrote the paper. All authors have read and approved the final manuscript.

## Conflict of Interest

The authors declare that the research was conducted in the absence of any commercial or financial relationships that could be construed as a potential conflict of interest.

## References

[1] Yang X Liang J Huang Q Xu H Gao C Long Y. Treatment of autoimmune glial fibrillary acidic protein astrocytopathy: follow-Up in 7 cases. Neuroimmunomodulation. (2017) 24:113–9. 10.1159/000479948 28922662

[2] Long Y Liang J Xu H Huang Q Yang J Gao C. Autoimmune glial fibrillary acidic protein astrocytopathy in chinese patients: a retrospective study. Eur J Neurol. (2018) 25:477–83. 10.1111/ene.13531 29193473

[3] Dubey D Hinson SR Jolliffe EA Zekeridou A Flanagan EP Pittock SJ. Autoimmune GFAP astrocytopathy: prospective evaluation of 90 patients in 1year. J Neuroimmunol. (2018) 321:157–63. 10.1016/j.jneuroim.2018.04.016 29793728

[4] Flanagan EP Hinson SR Lennon VA Fang B Aksamit AJ Pearse MP. Glial fibrillary acidic protein immunoglobulin g as biomarker of autoimmune astrocytopathy: analysis of 102 patients. Ann Neurol. (2017) 81:298–309. 10.1002/ana.24881 28120349

[5] Fang B McKeon A Hinson SR Kryzer TJ Pittock SJ Aksamit AJ. Autoimmune glial fibrillary acidic protein astrocytopathy: a Novel meningoencephalomyelitis. JAMA Neurol. (2016) 73:1297–307. 10.1001/jamaneurol.2016.2549 27618707

[6] Trau SP Gallentine WB. Autoimmune gFAP-Associated meningoencephalomyelitis: a Report of a pediatric patient. Pediatr Neurol. (2018) 82:50. 10.1016/j.pediatrneurol.2018.02.001 29622490

[7] Theroux LM Goodkin HP Heinan KC Quigg M Brenton JN. Extreme delta brush and distinctive imaging in a pediatric patient with autoimmune GFAP astrocytopathy. Mult Scler Relat Disord. (2018) 26:121–3. 10.1016/j.msard.2018.09.015 30245384

[8] Handoko M Hong W Espineli E Saxena K Muscal E Risen S. Autoimmune glial fibrillary acidic protein astrocytopathy following herpes simplex virus encephalitis in a pediatric patient. Pediatr Neurol. (2019) 98:85–6. 10.1016/j.pediatrneurol.2019.05.010 31248671

[9] Francisco C Meddles K Waubant E. Pediatric glial fibrillary acidic protein meningoencephalomyelitis: a case report and review of the literature. Mult Scler Relat Disord. (2019) 29:148–52. 10.1016/j.msard.2018.12.008 30885369

[10] Iorio R Damato V Evoli A Gessi M Gaudino S Lazzaro VD. Clinical and immunological characteristics of the spectrum of GFAP autoimmunity: a case series of 22 patients. J Neurol Neurosurg Psychiatry. (2018) 89:138–46. 10.1136/jnnp-2017-316583 28951498

[11] Li XJ Peng BW Hou C Liang HC Chen LF Zhu HX. A child of autoimmune glial fibrillary acidic protein astrocytopathy who had onset with meningitis. Zhonghua Er Ke Za Zhi. (2019) 57:882–4. 10.3760/cma.j.issn.0578-1310.2019.11.013 31665844

[12] Kunchok A Zekeridou A McKeon A. Autoimmune glial fibrillary acidic protein astrocytopathy. Curr Opin Neurol. (2019) 32:452–8. 10.1097/WCO.0000000000000676 30724768 PMC6522205

